# Experiments with Alloys

**Published:** 1898-10

**Authors:** J. D. Patterson


ARTICLE IX.

### EXPERIMENTS WITH ALLOYS.

DR. J. D. PATTERSON.

**At the American Dental Association at Old Point Comfort.**

I wish to say a word or two in regard to Dr. Crouse's very interesting paper. He says that the alloys upon the market do not exhibit the same peculiarities in different mixings. I wish to say that it is my opinion that alloys will exhibit the same results if they have been uniformly treated, and here is where the trouble is. Dr. Crouse, so far as appears from his statements before the association, at least, does not commence on a basis which gives any value to his experiment—that is, comparatively little value. So long as these different mixings of amalgam are made with different amounts of mercury and alloy, they will forever be different. He must commence on the basis of making the mixture with a definite amount of mercury and alloy, which is the proper amount, before these experiments will be of any value whatever. That is the statement I wish to make, and I think I am practically and scientifically right. Unless we can begin on that basis, what is the use of taking different alloys, mixed by different people, in a variety of ways, with different amounts of the materials, and experimenting with them?

It only proves what one man or another does, and gives us nothing of a scientific value. I have as much respect as any one for the experiments of Dr. Black, which have been alluded to, but when Dr. Crouse says that Dr. Black was the first one to make those experiments with the micrometer, he is certainly mistaken, because history tells us that many years ago Thomas Fletcher, F. C. S., of Warrington, England, and others made these experiments, or at least the important ones, on the same lines.

I am very much interested in alloys, and I am very much interested in having some settled and definite way of treating the various makes of alloy which produces a definite result. I think it can be done in the way I have indicated. Taking the alloys on the market, it is true that some of them require different amounts of mercury mixed with the mass of shavings or alloy—this difference, however, is slight; but let each be treated upon a scientific basis. When you find out the weight of alloy and mercury that make a perfect amalgam, you should adhere to that proportion. It should be the amount of alloy and mercury which, when put in a filling, under pressure, will give a harmonious relation, a perfect amalgamation, without any excess of mercury. When any excess of mercury is expressed, then the whole alloy is jeopardized, because it has been changed from what the manufacturers put out. I believe that the standard alloys are very carefully tested. I know that some of them are; not only every ingot, but different portions of every ingot, are tested thoroughly, and if they are not up to the standard, they are not sent out. One package acts the same, as compared with another, if it is treated the same. Of course, if the manufacturers take sufficient care, and if the materials are treated scientifically, on the basis I have spoken of, the results will be uniform. I have told Dr. Crouse in private, and I tell him here, that I will take, not twelve samples of the same amalgam, as he stated, but twelve times twelve, mix them, and put them in his forms, and they will practically give the same results under his instruments. I am ready to do that here, or at any time. The instruments with which I work are not here, or I would gladly do it now.—*Dental Brief.*

